# A Preliminary Study to Design and Evaluate Pneumatically Controlled Soft Robotic Actuators for a Repetitive Hand Rehabilitation Task

**DOI:** 10.3390/biomimetics7040139

**Published:** 2022-09-20

**Authors:** Claire Rieger, Jaydip Desai

**Affiliations:** Department of Biomedical Engineering, Wichita State University, Wichita, KS 67260, USA

**Keywords:** soft actuators, robotics, pneumatic, range of motion, unity, rehabilitation, microcontroller

## Abstract

A stroke is an infarction in the cortical region of the brain that often leads to isolated hand paresis. This common side effect renders individuals compromised in their ability to actively flex or extend the fingers of the affected hand. While there are currently published soft robotic glove designs, this article proposed a unique design that allows users to self-actuate their therapy due to the ability to re-extend the hand using a layer of resistive flexible steel. The results showed a consistently achieved average peak of 75° or greater for each finger while the subjects’ hands were at rest during multiple trials of pneumatic assisted flexion. During passive assisted testing, human subject testing on 10 participants showed that these participants were able to accomplish 80.75% of their normal active finger flexion range with the steel-layer-lined pneumatic glove and 87.07% with the unlined pneumatic glove on average when neglecting outliers. An addition of the steel layer lowered the blocked tip force by an average of 18.13% for all five fingers. These data show strong evidence that this glove would be appropriate to advance to human subject testing on those who do have post stroke hand impairments.

## 1. Introduction

Each year, nearly 800,000 people experience a stroke in the U.S. and in 2020, 1 in 6 deaths from cardiovascular disease was due to stroke [1,2], whereas an estimated 90% are left with some type of a disability [3]. Residual damage is so extensive that one study found about 65% of patients cannot incorporate an affected hand into their daily activities even six months after a stroke [4]. Thus, for therapy to be most effective, it must be initiated as soon as possible to include neuroplasticity stimulation, repetition, and consistency [5,6,7]. Soft robotic hand designs have increased significantly in the past 5 to 10 years with many improvements and unique innovative designs and implementations. However, publications involving soft robotic gloves can be traced back as early as 2005, T Noritsugu—Symposium on fluid power, who designed a pneumatic soft mechanism, a soft robot hand and a wrist rehabilitation device that have been developed as a wearable power-assist glove. Since then, new designs have included actuators that utilize restraint layers such as fiber wrapping [8,9,10,11], actuators made from geometric molds [12], gloves with double bladders for extension, gloves with torque compensating layers for extension [11], and much more. Starting in 2013, Polygerinos et al. developed multi-segment soft actuators that have expanding and bending segments using the fiber reinforcements [8]. This actuator used two inextensible materials. The first rubber layer molded around a hemi-circular steel rod. Woven fiberglass was glued to the flat face to serve as the strain-limiting layer. After molding the first rubber layer, fiber reinforcements were added to the surface and a Kevlar fiber was wound in a double helix pattern around the actuator. The wrapping was then encapsulated in a silicone layer. This publication also explored the analytical modeling of the explicit relationship between input pressure, bending angle, and output force. The geometry of the actuator’s inner chamber was evaluated within the analytical model and prototypes. Their analysis of actuator cross-sectional shapes compared rectangular (RT), circular (FC), and hemi-circular (HC) shapes using the same cross-sectional area. It was found based on analytical modeling that the hemi-circular shape was the most effective. The lower bending resistance showed the need for less pressure to achieve a larger curl and therefore the most effective shape for the purpose of the soft robotic glove. The glove design from Polygerinos et al. was able to achieve a maximum bending force tip of 8 N at a hydraulic pressure of 345 kPa. The weight of this glove was 285 g. This force was reported to be sufficient for rehabilitative hand positioning exercises but not for grasping and manipulating objects. The pressure required was notably higher than other designs, which is not energy- or cost-effective [10]. Wang et al. continued researching this using the same actuator design from Polygerinos discussed in the previous paragraph in 2016 [13], further modeling the design then testing the actuators with flex sensors. They improved the testing data for this design and documented the output force versus pressure at the varying fixed position angles of the actuator as well as the output of the force depending on the bending angle. They found that 180° required the most pressure and output the lowest force compared to 90°, which required the least pressure for the largest output of force. In 2016, Yap et al. released a soft robotic glove which only administered extension [14]. The pneumatic soft robotic actuators were fastened to the palmar side of the hand. This glove was able to administer 4.25 N of force in the direction of extension. This glove was designed for people experiencing clenched hand impairments [14]. In 2016, Yap et al. also published results for a fabric-reinforced pneumatic actuators with a corrugated top fabric layer. This design causes the top fabric layer to limit the radial expansion of the glove when pressurized. By using only silicone and fabric, the actuator was able to achieve a maximum bending tip force of 9.12 N at a lower pressure of 120 kPa. The fabric used had a modulus of elasticity or Young’s modulus of 0.5 N/mm [14]. Heung et al. designed a soft robotic glove with fiber wrapping, two actuator chambers, and two torque compensating layers made of A2 steel at the bottom to assist with extension. The two areas of flexion and extension were measured to match the PIP and MIP joints in the finger [11]. Their model includes the torque compensating layers of steel that are placed below the actuator. This model uses two actuator chambers that are placed of the MCP and PIP joints to focus on bending only in those areas and to require less pressure for smaller chambers [11]. In comparing the results for the MCP and PIP lengths for output angle as pressure increases, the data show an increase in output angle at given pressure for the longer lengths of air chambers. For these lengths tested, the longer the MCP and PIP air chamber length, the larger the bending angle that the actuator can accomplish at a given pressure [11]. There is a large variety of robotics that utilize extension; however, most of them are not soft robotic gloves. Instead, many of them are rigid robotic exoskeletons or prosthetics.

Studying the current designs, experimental analysis, and mechanical functions of current pneumatic gloves and related soft robotic technological publications, some extension mechanisms were identified. In the field of extension mechanisms, the other commonly implemented extension mechanisms are tendons. These are more common in rigid robotic exoskeletons than soft robotic exoskeletons. These utilize strings or chords to contract and expand the finger joints much like the biological tendons in the hand would. For example, Park, J. et al. produced a wearable finger actuating glove using surface-mounted tendon actuators designed for rigid robotic hands or prosthetics [15]. The actuator unit in this design involves a motor, motor holder, pulley, and two tendon strings for flexion and extension. This glove was designed to be combined with a “robot hand skeleton” to form a robotic hand. They evaluated the glove by the finger performing a touch sensitivity test where the strength was tested by exerting the highest force at the fingertip and measuring with a force sensor. The contact force was recorded at the rate of 1 kHz and the mean of the fingertip maximum contact force for five trials was 8.84 ± 0.23 N [15]. There have been few successful soft robotic gloves, however, that have been able to integrate the tendon mechanism into soft robotics because the strings need to be a soft flexible material. One publication utilized Kevlar threat, which is strong and flexible, and guided the thread through tunnels within to the glove along each finger. The tunnels were placed so that the flexion and extension of the finger could be facilitated when certain strings were tightened by the electrical motors attached at the end. Utilized an electrical motor pully system has a higher risk then utilizing silicone actuators and spring material such as sheet metal for motion because there is a risk of injury if the force is overexerted. Further, upon failure, the tendon model could risk locking the hand into certain positions whereas the flexible glove is a pliable material which does not hold any shape when unpressurized. Finally, other unique designs for extension include pneumatic and spring mechanisms seen in [14,16]. However, those designs do not align directly with the purpose of post-stroke paresis therapy as that designed for this paper. Yap et al. created a pneumatic extension mechanism geared towards the design of a Soft Robotic Glove for Hand Rehabilitation of Stroke Patients with Clenched Fist Deformity Using Inflatable Plastic Actuators. This glove was able to reach an extension torque of 1.03 Nm and did not facilitate flexion [14]. Park S. et al. sought to “present and evaluate transmission mechanisms by which exotendons can overcome hand spasticity for functional tasks with low motor forces and no rigid joints.” [16]. Their design consisted of springs attached on the distal and middle phalanx anchored at the back of the hand as pictured in the figure below. Additive manufacturing has shown great potential to fabricate parts or assemblies using hard or soft materials. Rapid mold fabrication, hybrid where additive and other manufacturing techniques used to design parts, and total additive manufacturing are three approaches being utilized in current field [17]. Soft monolithic pneumatic fingers were developed using a low-cost fused deposition modeling (FDM) 3D printer that generated a bending motion upon actuation [18]. The team of researchers were manufactured four-dimensional soft pneumatic actuators and monolithic bending pneuNets with embedded air connectors using additive manufacturing [19,20].

While there were many soft robotic gloves designed using pneumatic and hydraulic systems, most of them require higher pressure for actuation, do not offer both flexion and extension movements in one glove, and do not provide any range of motion (ROM) study that proves that whether the use of soft robotic actuators can closely mimic natural human finger movements for hand rehabilitation. The objective of this research paper was to design soft actuators that facilitate extension without compromising flexion in post stroke rehabilitation using less than 130 KPa pneumatic pressure followed by performing blocked tip force testing on newly fabricated actuators prior to conduct three ROM studies on ten participants without hand dysfunction. These studies demonstrated the effect of the extension steel layer and the achievable bending angle in active unassisted ROM versus passive assisted ROM.

## 2. Materials and Methods

### 2.1. Soft Actuator Design and Fabrication

Optimizing a soft pneumatic glove with steel ribbons requires two general categories be taken into consideration. These include those to satisfy the technical parameters and to satisfy the practical requirements. To satisfy the technical parameters, this glove needs to reach or exceed the standards set by previous designs discussed in the Literature Review. Technical parameters include the actuation pressure required for of the actuators, the output flexion force, and achievable ROM. To satisfy the practical components of this design, cost, comfort, and ease of use, materials research and live testing must be completed. According to Polygernios et al. the flexion force needed to manipulate objects of daily living is between 10–15 N [8]. For rehabilitative purposes, as this design is geared towards, the required amount of force would be less since the goal is to simply bring the fingers through ROM. However, depending on the time passed since the stroke incident, patients can have spasticity and stiffer joints. Therefore, it is hard to estimate the force needed since it will vary with each participant. It is estimated that the force required for a patient in the flaccid hand state would require the same amount as an unimpaired person’s hand at rest. A patient receiving therapy directly following the indecent would begin in this flaccid state as discussed in the introduction.

Practically, the glove needs to be cost effective and easily fabricated. For both practicality and user friendliness, the glove needs to be transportable and light weight. Most soft robotic gloves follow a similar base design. Generalized, this design consists of a soft robotic flexible and expandable air chamber with a constraining material fastened to one part of the actuator. As the actuator is pressurized, the unrestrained part of the actuator expands while the non-expandable part restrains linear expansion, producing a bending motion in the geometry. The design choices in this work consisted of the materials used, the shape and length of the actuators, the geometry of the air chamber. Finally, this design needed to implement a method of returning the fingers to extension after having been curled.

**Figure 1:**
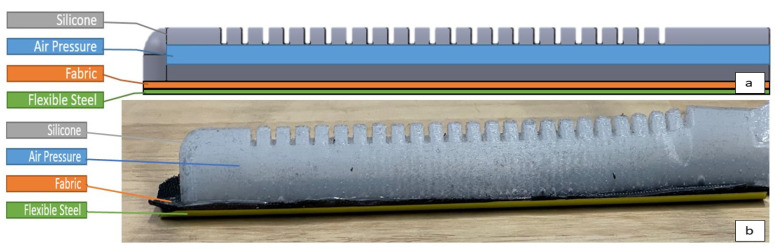
Diagram showing the make-up of the layers of the soft robotic actuator finger design (**a**) and actual fabrication (**b**).

The final design consisted of a nylon fabric glove with actuators attached to the five fingers of the glove. There were three important layers for each of these attachments. The layer lowest, closest to the dorsal side of each finger, was the flexible steel layer. This was a thin strip of flexible stainless steel curved upwards lateral-medial wise. This layer was attached via a pocket and therefore removable or interchangeable. The steel layer served the purpose of promoting extension of the finger after flexion. The next layer up was a fabric layer embedded into the actuator with silicone. This layer was one way stretch nylon fabric and served the purpose of restraining the silicone on the bottom surface of the actuator to constrain expansion on the bottom layer. On top of the fabric was the silicone actuator with a semi-circular air chamber inside. The actuator creates the flexion motion of the finger when actuated. Figure 1 shows the conceptual design (a) and actual fabrication (b) of the actuator, respectively.

The material used for the actuator body in the mold was Dragon Skin 10 silicone as used in [21] due to the cost and simplicity of implementation. Dragon Skin 10 silicone is a very strong and stretchy material with high elasticity. Dragon Skin 10 has a hardness of 10 A which is the lowest Dragon Skin produced. Dragon Skin is also skin safe and does not require a vacuum chamber or heating to cure. Silicone is a very elastic material and therefore can be deformed into a new shape and return to its original shape and size when the force causing the deformation is removed. The force causing deformation in this case is air pressure, which inflates the chamber inside the actuator body and causes the actuator to expand [22]. Similarly, the resistance material added to the bottom layer of nylon coated flexible steel also returns to its original shape after deformation of a certain axis. Similar to the steel found in snap bracelets and measuring tapes, the steel layer snaps back to a straight sheet when bent at any point. The geometry of the sheet of metal used also has a U shaped curve which adds to the stiffness and elastic force which drives the metal back to the straightened position.

Retractable reels that coil back in when extended were considered but places tension on the string when curled were considered as well but a reliable way to connect a string to the tip of the finger was not discovered. Due to the nature of the coil, the force is transferred to tip of the finger rather than even distributed along the length of the finger as with the flexible stainless steel. This force distribution could result in injury and discomfort whereas the flexible stainless steel is a smaller force load that is distributed and brings the finger back into place in a slower and more controlled manner. Retractable reels that coil back in when extended were considered, but the recoil created too much force in a short period of time. Due to the nature of the coil, the force is transferred to tip of the finger rather than even distributed along the length of the finger as with the flexible stainless steel. This retractable reel force distribution could result in injury and discomfort whereas the flexible stainless steel is an evenly distributed smaller force load which returns the finger back into place in a slower and more controlled manner.

**Figure 2:**
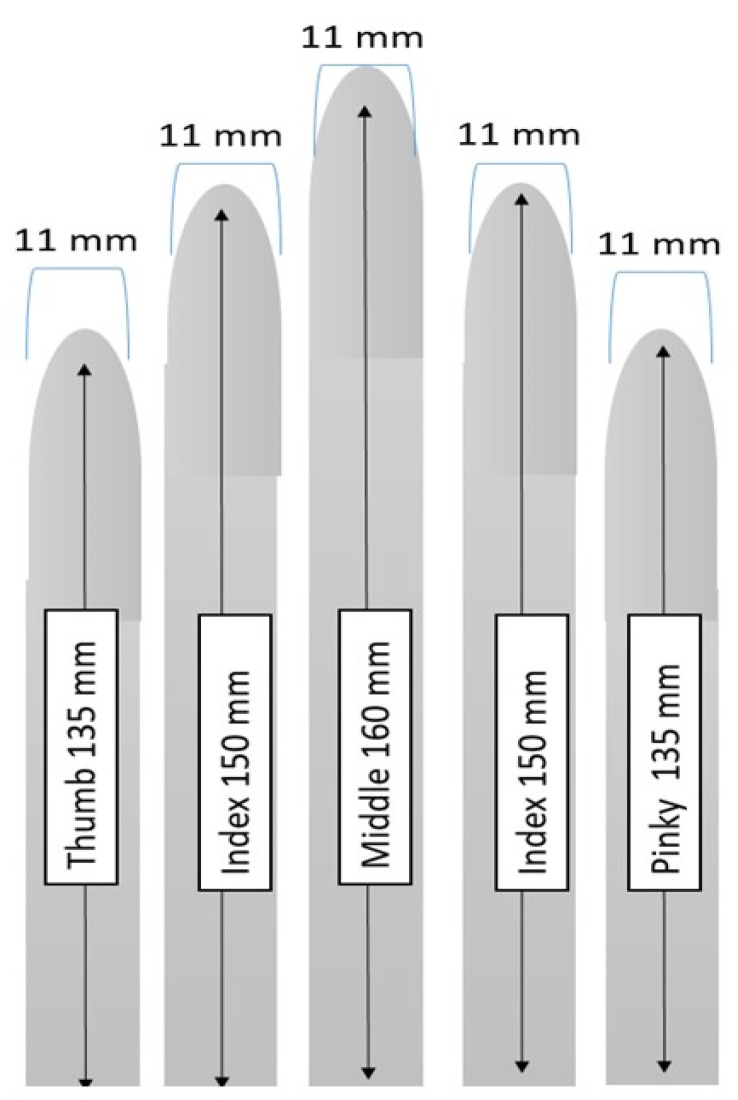
Dimensions of height and width of the actuators for each finger.

Due to the condition of post stroke patients, the return motion of the hand needs to be slow and gentle. If therapy moves the muscle too quickly or forcefully, it can elicit abnormal muscle tone which can cause more spasticity [23]. The silicone actuators were based around the design of Gerges et al. [12] due to the efficacy of Gerges’ published results and easily accomplished fabrication mold design. The actuators were dimensioned to mimic the length and width of average human fingers as shown in Figure 2.

**Figure 3:**
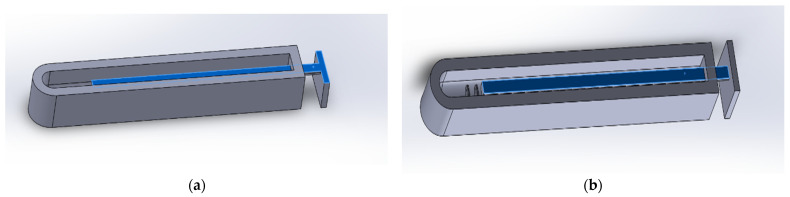
Mold rod design modified (**a**) compared to rod design (**b**) used in the literature [12].

The mold was for the silicone actuator was altered from the design of Gerges [12]. The inner rod was re-designed so it could be 3D printed lying flat rather than upright as shown in Figure 3a,b, respectively. This allowed the rod to be bendable due to the long length of the 3D printed layers. This made the process of removing the rod after the solidification of the silicone much more achievable. This also made the rod easier and faster to 3D print.

**Figure 4:**
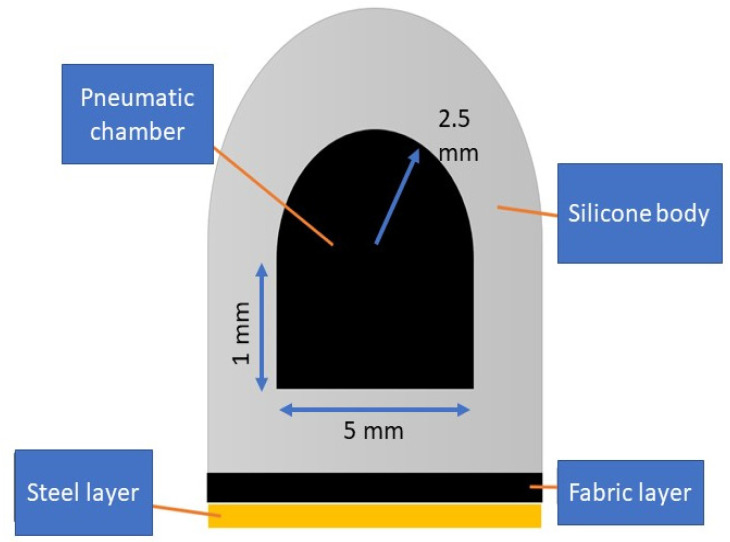
Diagram of the cross-sectional area of the actuator including the fabric and steel layer at the bottom.

This design uses a pneumatic chamber rod which has a combined semi-circle and rectangle cross-section based on the design from Gerges [12]. The semicircular cross section was selected based on results from Polygerinos et al. [8]. Figure 4 shows the diagram of the cross-sectional area used during fabrication process.

Once the mold for the actuator body and pneumatic chamber manufactured using 3D printing, the Dragon Skin 10 medium including the curing agent poured into the mold with a rod. A polyester fabric was placed on the top and the entire setup was placed in a room temperature for 24 h followed by demolding process to extract newly fabricated actuator from the mold. A Sil-proxy glue was then used to connect air tube with pneumatic chamber inside the actuator. At the end, all the actuators for each finger was hand stitched on a glove. A flexible steel layer was then placed with the cloth glove and the actuator. Table 1 shows the entire fabrication process executed prior to testing. Overall, it takes approximately 24–30 h to fabricate the pneumatic soft actuator including the time to print the molds and the estimated cost for a single actuator is calculated to be around $24 which includes 3D printing charges, PLA material, Nylon cloth, tubes, and Dragon Skin 10 material.

### 2.2. Blocked Tip Force Testing Setup

**Figure 5:**
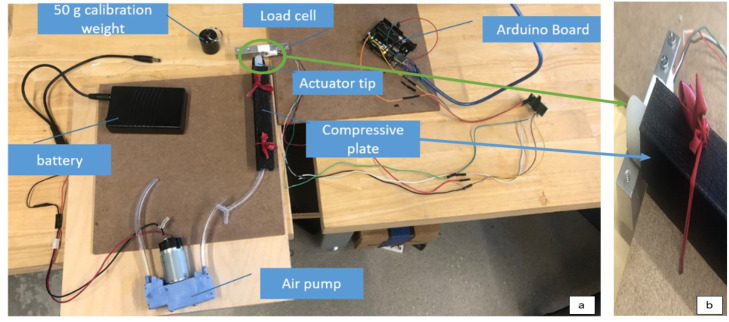
Apparatus showing the experimental set up of blocked tip force measurements using load cell (**a**) and close up of the blocked tip force apparatus (**b**).

To measure the output force of the glove, the blocked tip force (BTF) measurement was obtained for each of the geometries of the pneumatic chambers as well as the final design with and without the flexible stainless steel for each length of finger. The standard measurement for soft robotic actuators is the blocked tip force, or BTF to test their force output abilities [14,24]. This allows measurement and comparison of the actuator’s ability generate sufficient force and torque to assist in finger flexion. The BTF is a measurement of the force produced at the tip of the actuator when pressurized. Figure 5a shows the entire BTF setup used in this research. The BTF measurement prevents the actuator from creating a bending motion to maximize the pressure at the tip where it would be distributed throughout the glove. In this apparatus, the actuator was placed between a compressive plate on top of it and flat surface below. The compressive plate was placed on top of the actuator and tied down to keep the actuator from inflating and bending anywhere except for the exposed distal tip which is allowed to protrude off the bottom surface as shown in Figure 5b. Underneath the protruding tip of the actuator was the load cell. The transducer load cell had a limit of 20 kg and was connected using an Arduino Uno board. The load cell converted the force output by the tip of the actuator into a weight reading in grams. This was standardized through a calibration.

For every actuator trial, the load cell was re-calibrated to a 50 g weight. Once the calibration was complete, the actuator was placed on a flat platform with the tip touching the load cell sensor. The actuator was constrained so only the tip of the finger would bend. The output force was documented continuously every 0.1 s on the serial monitor measured once the air pressure was started. To convert the output of g to newton, it was assumed there was no acceleration acting upon the force by using the stable. The data collected were averaged over the period of time when the force had stabilized allowing the neglect of addition acceleration outside of gravity. Within the data analysis, the output in grams from the load cell was multiplied by 0.0098066 to convert the grams to newton using gravity. The data were analyzed to find the peak forces output within each pressurization of the actuator for each trial. There were six trials for each actuator size, three without the stainless flexible steel layer and three with. One trial consisted of the actuator starting at 0 kPa and being pressurized to up to 150 kPa while all parts of the actuator were blocked from expanding except for the tip. The overall maximum value and average peak value over the three trials was found for each of these experimental sets of data. To further evaluate the actuators, two actuators of equal length but larger chamber were tested and compared without involving the stainless-steel layers. Three trials were taken for each of these actuators pressurized force output as well and compared in the results section. The index and ring finger actuators have the same dimensions and therefore were grouped into one trial.

### 2.3. Human Subject Testing

The purpose of the human subject testing was to execute a feasibility study towards the efficacy of the glove within real life applications. Specifically, the implementation of the extension metal layer was evaluated to test if it outputs a sufficient extensive force to return the participants hand to perform repetitive motion therapy. To implement human subject testing, an Institutional Review Board (IRB) application was submitted and approved (#5258) before testing. The testing parameters included only test subjects without history of neuromuscular disability, stroke, hand injury, or hand impairment between 18 to 60 years of age. The participants placed their wrist at a neutral support position over a raised platform where their hand hung off the edge of the platform. Their hands were kept in pronation for all trials and instructed to leave their hand at rest when not actively moving their finger during the ROM trials. The testing consisted of five trials. These five trials were executed in varying order as to remove influence involving the fatigue or stretching of the participant’s hand during the trials. In each trial, a Unity code was utilized to provide prompts or input values to the control system in which each of the five fingers was prompted five times in random order.

To ascertain the optimal position for testing 2 priorities were considered. The first was maximizing test results and the second was replicating the situation in which the pneumatic glove will foreseeably be used by post stroke patients. Finger movements occur around two main axes: flexion/extension and abduction/adduction [25]. Flexion is the act of bending or state of not straightened while extension is the opposite an unbending movement or straightened position [21]. Abduction is movement of a body part away from the median plane and adduction is the movement towards the median plane [21]. In addition to flexion/extension, abduction/adduction the thumb has an additional movement. The movement of opposition/reposition which mainly offers the hand an increase in grasping and manipulating a variety of objects [26].

The subject’s wrist was positioned in neutral to maximize a resting position and assist in allowing fingers to flex and extend independently of one another. The movements of the forearm are pronation/supination and are completed by the radio-ulnar complex, influenced by some specific shoulder positions. Supination, palm side facing the ceiling with the elbow flexed, was not as desirable an option as it may have impeded the pressure of the flex actuators. Pronation was also preferred as healthy hands approach most objects in pronation to grasp it.

To maximize the accuracy and consistency of the test results subjects were tested seated with their right shoulder slightly abducted and their elbow flexed resting on a box on a chair shown in the figure below. This combination of relaxed positions allowed test subjects to sit comfortably in a side chair and assisted in isolating the desired movements of individual finger flexion and extension. In anticipation of the eventual user, a post stroke patient the process of motor recovery was considered.

Stroke patients initially experience flaccid muscles, followed by the development of patterned muscle movements, or synergies [5,6,27,28]. Synergies could be defined as a spatial configuration of the hand shape that is common across the various tasks [6,25]. These synergies are how the affected hand relearns purposeful movements. As spasticity develops patients are drawn into various degrees of abnormal hand flexion and pronation making it difficult to extend fingers, hence the preferred position of finger flexion and forearm pronation. Ideally the pneumatic glove should replicate normal movement.

The metacarpal heads were situated along the outer ridge of the box to allow space for the fingers to flex. Testing consisted of randomized isolated pressure applied to the dorsal side of an individual digit. Therefore, it was necessary to provide enough space to allow digits to move independently. All positions were chosen to maximize comfort and facilitate relaxation as subjects were asked to allow the pneumatic glove to provide all hand movement.

**Figure 6:**
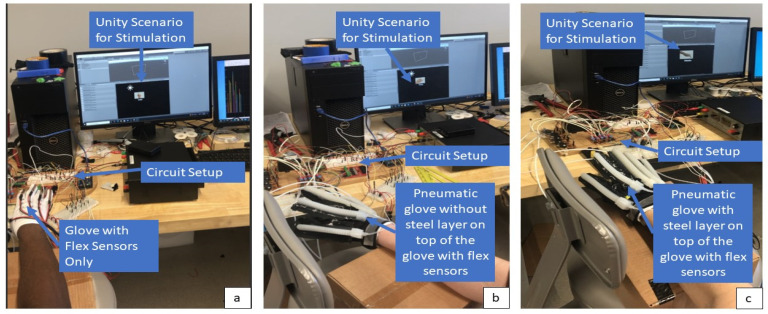
ROM testing for human subject testing where the subject actively flexed their finger withouth the assistance of the pneumatic pump according to the Unity promt on the screen. The angle of flexion was recorded using flex sensors in three conditions of no pneumatic glove (**a**), pneumatic glove without steel ribbon insert (**b**), pneumatic glove with steel ribbon inserts (**c**).

There were three ROM trials and two pneumatic trials. In the ROM trials, the pneumatic pump was kept off and the participant was told to flex the prompted finger to the furthest to the furthest of their capabilities while remaining in their range of comfort. The participant was sat in front of a screen with their hand in pronation on the raised platform in which these prompts were given for 4 s with 4-s-long breaks in between prompts. The first ROM trial was with only a nylon glove with flex sensors attached to record the angles as shown in Figure 6a. For the second and third ROM trials, the pneumatic glove was placed over the flex sensor glove to test the restrictions the pneumatic glove placed on the ROM. The second ROM was the pneumatic glove without the steel inserts (Figure 6b) and the third was without the steel inserts (Figure 6c).

## 3. Results

Upon successful completion of the soft actuator fabrication process, blocked tip force testing (Section 3.1) is considered the best method to evaluate the soft actuators prior to human subject testing. This section demonstrates the effect of adding/removing the steel layer by acquiring blocked tip force followed by discussing ROM study on three different conditions (Section 3.2) listed as no pneumatic glove, with pneumatic glove without steel, and with pneumatic glove with steel layer. Each participants’ subjective feedback on comfort (Section 3.3) was acquired at the end of the experiment.

### 3.1. Blocked Tip Force Testing of Fabricated Soft Actuators

**Figure 7:**
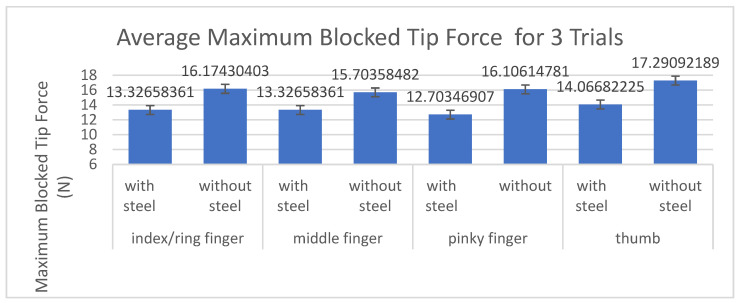
Comparison of blocked tip force per actuator of each finger with and without the layer of stainless flexible steel.

Each actuator was tested three times during the blocked tip force testing. Figure 7 shows the average maximum blocked tip force acquired over three trials and the comparison of with and without steel layer with respect to maximum blocked tip force in Newtons (N). The index/ring fingers were tested as one actuator because the mold dimensions for those actuators were the same. The middle showed the smallest change in blocked tip force at 15.14% percent decrease when adding the steel while the pinky showed the largest difference with a 21.13% difference.

Table 2 showed for the Blocked Tip Force Measurements that the addition of the steel layer lowered the blocked tip force by an average of 18.13% for all five fingers.

### 3.2. Range of Motion Testing on Human Subjects

In testing the ROM, the baseline (no pneumatic glove condition) data showed, on average, the ROM was smallest at the pinky. Table 3 shows the average maximum angle acquired in degrees from 10 participants. In order from smallest average ROM peak angle achieved to greatest, the order went from pinky at 52°, middle at 68°, ring at 79°, index at 87°, and thumb at 91°. The pinky showed the least variation in the 3 conditions tested whereas the pinky showed the largest variation in the 3 conditions results. In comparing the differences between the average ROMs accomplished in the three different conditions for the unassisted trials, the thumb, index, middle, and ring show a variance within 10° or less for all three conditions. The pinky shows a variance of 34° between the trials.

**Figure 8:**
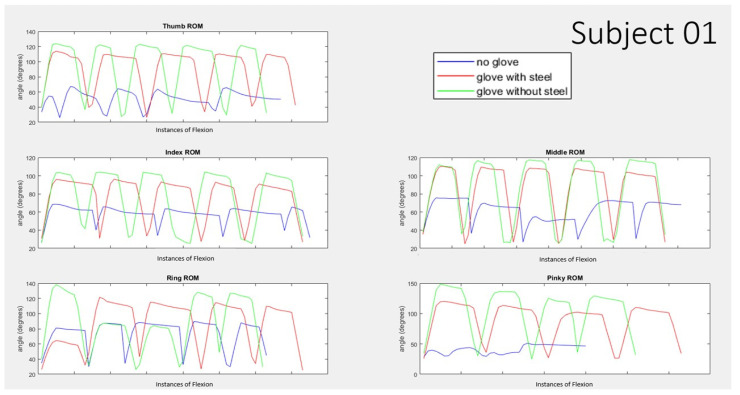
Range of Motion for 5 nonsequential instances of participant actuated finger curling with no pneumatic assistance for 3 different conditions of wearing no pneumatic glove (shown in blue), wearing the pneumatic glove with steel inserts (shown in red), and wearing the pneumatic glove without the steel (shown in green) for subject 01.

**Figure 9:**
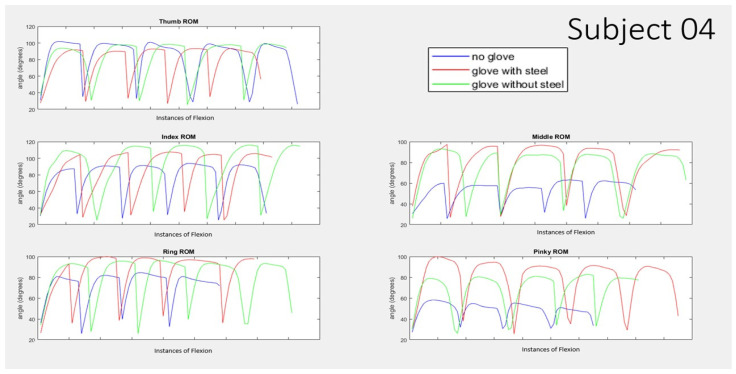
Range of Motion for 5 nonsequential instances of participant actuated finger curling with no pneumatic assistance for 3 different conditions of wearing no pneumatic glove (shown in blue), wearing the pneumatic glove with steel inserts (shown in red), and wearing the pneumatic glove without the steel (shown in green) for subject 04.

The graph below shows the angle reading from the flex sensor of the five nonsequential trials for each finger for subject 01 (Figure 8) and subject 4 (Figure 9) comparing 3 cases of no pneumatic glove, with pneumatic glove with steel, and with pneumatic glove without steel. For the purpose of visual comparison for the results of each individual finger, the five nonsequential instances have been placed alongside one another. These two sets of data showed the largest and smallest differences between the conditions consistently. Subject 01 consistently shows a trend of “no glove” condition shown in blue being about 40° or more lower than those trials with the pneumatic glove shown in red and green. Subject 04, in contrast, consistently a trend of “no glove” condition shown in blue being about 20° or less lower than those trials with the pneumatic glove shown in red and green for the thumb, index, and ring finger. Subject 04 shows a larger decrease for no glove condition for the cases of the middle finger and pinky finger.

**Figure 10:**
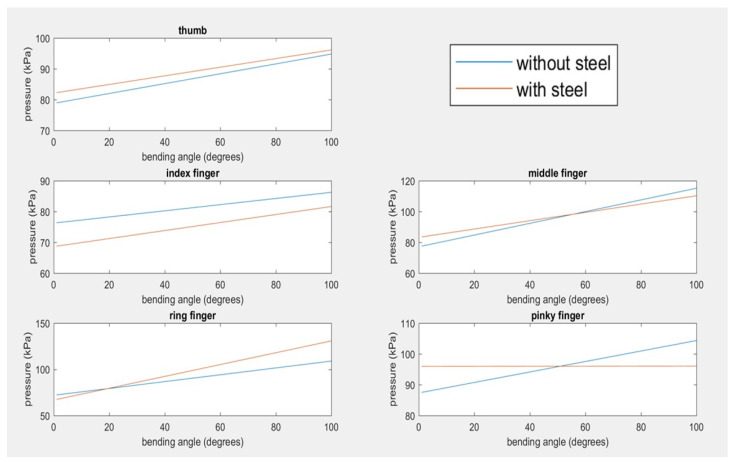
Best fit line of angle vs. pressure for 10 subjects using averaged point-slope intercept m and b values for five the fingers.

In comparing the best fit line of angle vs. pressure using averaged point-slope intercept m and b values for all the subjects shown in Figure 10, the slopes all follow a positive trend of increasing pressure results in increasing angle. The thumb, index, middle, and ring finger produce lines for with and without the steel that stay within about 10 kPa of one another for any given angle. The pinky is an outlier in this trend, as the ‘with steel’ trial produced an almost flat line slope of 0.008. Another comparison of the pneumatic conditions for with and without steel were the peak angles achieved for each condition during their trials as displayed in the table below.

Table 4 compares the average peak angle and the associated pressures by finding the percent difference. For the thumb, middle, ring, and pinky finger, the “without steel” produced a larger average peak angle than the “with steel” conditions. However, the “with steel” peaked at a lower pressure for those cases. Following same trend as the other data, the pinky is an outlier in the case of Table 5 comparing the cases of with and without steel where the percent differences for the pinky angle is 24% with a very small difference in pressure of −1.85%. The middle finger in this table also shows a significantly larger angle difference in comparison to the thumb, index, and middle finger; however, the associated pressure is also significantly increased and therefore follows the trend of the other fingers.

On average, the pneumatic actuations without the steel layer reached a peak angle that was 7% higher than the participants baseline ROM. The pneumatic actuations with the steel layer reached an average peak angle that was 7.2% lower than the participants baseline ROM.

### 3.3. Participants Comfort Ratings

Upon the end of their testing, the participants were asked to complete a brief survey about their experience with the glove. Table 6 displays the average rating given to each category.

The participants were asked to give an overall score of comfort of the glove, in which 4.4 was the averaged answer. When asked to rate “Is the soft robotic glove heavy? On a scale of 1–5 where 5 is the heaviest” the participants replied with an average score of 3.8. At the end of the survey was an optional section that allowed participants to provide any other feedback or comments they had on this study. The comments included one note that the participant thought their fingers were too short for the glove to give accurate results. No other significant comments were made.

## 4. Discussion

The results in testing the tip force output of the load cell performance showed that the addition of the flexible steel layer creates a resistance that reduces the bending force of the actuator. This reduction, however, is not significant enough to take the bending force created outside the range of sufficient force. The estimated necessary force to curl a finger range about 10–12 newton [21]. Measurements of the averaged maximum blocked tip force ranged from 12.7–14.1 N with the layer of steel and 15.7–17.2 N without the steel.

In evaluating the blocked tip force measurements in Table 1 and Figure 7, there is a trend between length of actuator and the percent decrease in blocked tip force when adding the layer of stainless steel. In order of length, the pinky and thumb are the same length and the shortest of the three lengths created. Therefore, the largest difference in blocked tip force between the trials with and without the steel occurred in the two shortest actuators, the thumb and pinky. The smallest difference in blocked tip force between the trials with and without the steel occurred in the longest actuator, the middle finger.

In the design of this study, it was known that the target audience this glove is designed for would not be within the qualified pool of participants. The study participants averaged an age of 38, ranging from age 23–60. According to Stanford health care, most stroke patients are older than 65 years of age. While this glove is designed for those with hand impairment, no eligible participants were allowed to have a history of hand injury, neuromuscular issues, or stroke paresis. While this population does not accurately represent the pool of people this glove is designed for, this study provided a strong baseline of data in investigating the efficacy and limitations of the glove when put into practice on varying hand sizes, strengths, and other characteristics. Furthermore, utilizing participants without hand impairment allowed for the opportunity of comparing the pneumatic gloves ROM to that of an unimpaired person’s baseline ROM. The baseline ROM data showed the thumb having the greatest ROM in Table 2. This was expected due to the anatomy of the hand. The thumb’s metacarpal bone is connected via a saddle joint whereas the other fingers are connected via hinge joints. This allows the thumb to have a larger ROM. It is also regularly used in everyday tasks and therefore the flexion of the thumb is a familiar movement for most people.

On the contrary, the dramatic difference in ROM is not as explicable based solely on anatomy. While the pinky is the smallest and less used in daily tasks for individual flexion, the ROM is relatively comparable to that of the other hinged fingers [28,29]. One possible explanation for the very small ROM recorded for the pinky finger is due to the flex sensor placement. Since the pinky tended to be shorter than the length of the glove, the flex sensor usually extended beyond the edge of the pinky. The flex sensors were found to give stronger signals if bend at the tip. For one subject, the pinky flex signals were not showing up at all until the glove was pulled all the way down and fixed at the wrist to keep the pinky reaching the end of the flex sensor. It was noted this subject had small hands and thin fingers for the size of the glove. While the glove was pulled as far down the wrist as possible for each subject, the webbing between the index, middle, and ring would usually restrain the glove before the pinky could fill the pinky hole in the glove. The gloves spaces for the index, middle, and ring were usually shorter than the length of the finger while the pinky was longer. The tightness of the glove could also affect the results as some people have wider fingers than others. In the case of thinner fingers, the finger would have more space to move within the glove before pulling the fabric taught and moving the flex sensor.

The length issue of the glove on the pinky also provides an explanation for the dramatic change in ROM seen in the pinky in when the pneumatic gloves were added. Since the silicone actuator provides a stiff material over the flex sensor which matches its length, the actuator causes the tip of the flex sensor to bend when it is flexed increasing the angle reading of the flex sensor.

The data of subject 01 vs. subject 04 display the large differences in ROM per subject occurring within the human subject testing. Factors for these differences could include hand size, finger lengths, ROM, and effort on part of the subject. In comparing the best fit line of angle vs. pressure using averaged point-slope intercept m and b values for all of the subjects shown in Figure 10, the slopes all follow a positive trend of increasing pressure results in increasing angle. This trend is comparable to the publications in [8,11,12,24] and was expected given the design of the actuators. The thumb, index, middle, and ring finger produce lines for with and without the steel that stay within about 10 kPa of one another for any given angle showing that there is not a significant difference in the amount of bending angle achieved for a given pressure. The outlier in these data in the pinky finger. The ‘with steel’ trial produced an average slope of 0.008 which was practically a flat line. This would indicate there is no increase in angle given an increase in pressure; however, the flat line occurs at a high pressure near 100 kPa. Therefore, this instead indicates the angle has already achieved a high value by the time the data begin to record the pressure and angle values. This could be attributed to the same issue that was occurring with the ROM trials where the edge of the pinky glove is not filled by the finger length. The empty fabric at the tip of the glove is extremely easy for the glove to bend with a very small amount of pressurization. As such, the angle jumps to a high value with very small amounts of pressure and the recording only displays data after that quick jump. The steel layer exaggerates that effect immediately taking the small force and jumping to a 90-degree angle due to the geometry and material of the steel layer.

Most importantly, the steel layer was able to actively assist the extension of the glove while minimally impairing the glove’s ability perform flexion motions and reach a sufficient flexion angle. The results from the human subject testing showed a consistently achieved an average peak of 75 or greater for each finger while implementing the steel layer.

Finally, the proposed design was able to be compared to other publications previously discussed in the literature review. This allows for a direct appraisal of the resulting output of the glove’s performance. Table 7 shows various publications of soft robotic glove designs in comparison to this design, referred to as Rieger and Desai 2022 in the last row.

## 5. Conclusions

Through prototyping, evaluation, and unimpaired human subject testing, the proposed design has shown sufficient potential and promising results to offer repetitive hand rehabilitation tasks. The added steel layer was able to assist the extension of the glove while minimally reducing the glove’s ability to perform flexion motions. The blocked tip force results ranged from 12.7–14.1 N for the different-sized actuators for each finger and these actuators were able to perform flexion movement using 120 KPa pneumatic pressure. Human subject testing on 10 unimpaired participants showed that the actuators reached a sufficient force and flexion angle during testing. Overall, this preliminary study indicated that the use of an added steel layer in the glove allowed participants to extend their fingers when the air goes out of these actuators, but it slightly reduces their ROM. An average peak of 75° or higher was achieved for each finger movement using the steel layer, which further justifies the benefits of providing flexion and extension movements in one glove and would increase the adaptation rate of this technology among stroke patients as well as offer faster recovery by performing flexion and extension movements. Further study would need to be conducted on people experiencing hand paresis or impairments to obtain accurate results on the efficacy of the proposed glove.

## Figures and Tables

**Table 1:** Fabrication process steps.

Fabrication Steps	Fabrication Process
1	Actuators body molds and pneumatic chamber rods were designed using Dassault Systemes’ SolidWorks 2020 version.
2	Additive manufacturing used to 3D print molds and chamber rods using Polylactic acid (PLA) material. This material is easily available and not expensive. It is also supported by most of the 3D printers.
3	Dragon Skin 10 Medium, liquid silicone, was poured in the 3D printed finger molds with pneumatic chambers. This liquid silicon allows curing process which converts liquid silicon into stretchable solid form.
4	A polyester fabric layer was placed on the top that allows bending motion when pressurized. Nylon material was used.
5	The setup then placed in a room temperature for approximately 24 h prior to demolding the fabricated soft actuator.
6	Gently removed the pneumatic chamber rod from the actuator which makes the proximal area of the fabricated soft actuator open.
7	The fabricated actuator then placed vertically with an open end submerged in a small cup filled with the same liquid material for closing its end.
8	A small drill was used to create a hole at the proximal end to access the pneumatic chamber which connect with air tubes.
9	Sil-Proxy, glue for silicone, was then used to seal the connection between the air tube and the pneumatic chamber.
10	A flexible steel layer was placed between the cloth glove and the fabricated actuator followed by performing stitching around the sides.

**Table 2:** Percent decrease in blocked tip force when adding steel layer.

	Middle	Index/Ring	Thumb	Pinky	Average All Fingers
Percent decrease in blocked tip force when adding steel layer	15.14%	17.61%	18.65%	21.13%	18.13%

**Table 3:** Average maximum angle in degrees for 10 subjects’ ROM trials comparing 3 cases of no pneumatic glove, with pneumatic glove without steel, and with pneumatic glove with steel.

Active Unassisted ROM Peak Angle Data			
		Average Angle (Degrees)	Standard Error (Degrees)
Thumb	no pneumatic glove	91.03	5.05
	with pneumatic glove without steel	92.33	5.40
	with pneumatic glove with steel	96.18	4.10
Index	no pneumatic glove	87.20	5.61
	with pneumatic glove without steel	91.54	4.83
	with pneumatic glove with steel	97.39	3.53
Middle	no pneumatic glove	68.60	5.48
	with pneumatic glove without steel	76.62	6.24
	with pneumatic glove with steel	74.49	6.21
Ring	no pneumatic glove	79.01	6.83
	with pneumatic glove without steel	83.96	6.94
	with pneumatic glove with steel	82.51	7.16
Pinky	no pneumatic glove	52.40	3.78
	with pneumatic glove without steel	82.79	8.38
	with pneumatic glove with steel	86.25	7.34

**Table 4:** Average peak angles and associated pressure values averaged for 10 subjects for each of the 5 fingers compared between 2 conditions of pneumatic actuator with and without the steel layer.

Peak Angles and Associated Pressures for 10 Subjects							
		Average Values for Peak Angle, Associated Pressure, and Standard Error				% Difference of with vs. without Steel	
Finger	Condition	Angle (Degrees)	Standard Error for Angle	Pressure (kPa)	Standard Error for Pressure	Angle (%)	Pressure (%)
Thumb	without steel	61.17	3.97	92.27	2.32	3.63	−4.79
	with steel	58.95	4.43	96.69	3.07		
Index	without steel	85.80	4.12	81.98	4.11	−1.42	1.46
	with steel	87.02	5.39	80.78	2.71		
Middle	without steel	64.81	4.92	95.71	1.64	6.71	−2.37
	with steel	60.46	4.39	97.98	3.00		
Ring	without steel	69.70	6.86	90.90	2.23	20.29	−8.24
	with steel	55.56	5.22	98.39	2.01		
Pinky	without steel	97.97	6.69	96.28	2.69	24.36	−1.85
	with steel	74.11	5.89	98.06	1.84		

**Table 5:** Percentage of “no glove” condition ROM met by peak angles during pneumatic actuation Trials with and without steel layer.

Finger	Condition	Percentage (%)
Thumb	without steel	67.20
	with steel	64.76
Index	without steel	98.40
	with steel	99.79
Middle	without steel	94.48
	with steel	88.13
Ring	without steel	88.22
	with steel	70.33
Pinky	without steel	186.97
	with steel	141.44
Average	without steel	107.05
	with steel	92.89
Average not including pinky	without steel	87.07
	with steel	80.75

**Table 6:** Averaged survey results of subjects when asked to rate the pneumatic glove on comfort level where 5 is the most comfortable and 1 is the least.

Participants’ Comfort Rating of Glove						
	Flexion		Extension		At Rest	
	Average Score	Standard Deviation	Average Score	Standard Deviation	Average Score	Standard Deviation
Thumb	4.50	1.08	4.6	0.84	4.7	0.67
Index	3.90	1.6	3.8	1.55	4.2	1.48
Middle	4.5	0.97	4.4	1.07	4.3	1.25
Ring	4.1	1.2	4.5	0.97	4.2	1.48
Pinky	4.5	1.08	4.9	0.32	4.6	1.26

**Table 7:** Table comparing the characteristics and results of similar soft robotic glove designs and publications.

Author and Reference	Year Published	Tip Force Output	Pressure Required (kPa)	Weight	Extension Method
Polygerinos et al. [10]	2015	8 N	345	285 g	none
Wang et al. [13]	2016	8 N	345	285 g	none
Zhao et al. [30]	2016	5 N	270	/	none
Yap et al. [31]	2017	9.12 N	120	180 g	none
Heung et al. [11]	2019	/	200	207 g	Torque compensating layer
Gerges et al. [12]	2019	9.5 N	180	120 g	none
Chizik et al. [32]	2021	14 N	120	196 g	Spring layer of metal
**Rieger and Desai**	**2022**	**12 N**	**120**	**149 g**	**Steel layer**

## Data Availability

Not applicable.
